# The Experience of Using Urinary Liver-Type Fatty Acid-Binding Protein for Follow-Up of Toluene Poisoning: A Case Report

**DOI:** 10.7759/cureus.50438

**Published:** 2023-12-13

**Authors:** Hironari Fujikawa, Ryo Ichibayashi, Tomoka Sato, Momoko Shimizu

**Affiliations:** 1 Internal Medicine, Toho University Sakura Medical Center, Chiba, JPN

**Keywords:** liver-type fatty acid-binding protein, bicarbonate stress test, chronic toluene toxicity, toluene poisoning, renal tubular acidosis

## Abstract

Toluene poisoning is diagnosed based on toluene exposure history and the level of hippuric acid in the urine. Regular blood and urine tests are performed for follow-up.

A 54-year-old man collided with a utility pole while driving a car and was rushed to our hospital with a complaint of loss of consciousness. Although the trauma was minor, toluene poisoning was suspected based on the presence of impaired consciousness, occupation is a painting job that involves toluene, and the presence of metabolic acidosis of the normal anion gap. Urinary hippuric acid and urinary liver-type fatty acid-binding protein (L-FABP) were measured, and a diagnosis of renal tubular acidosis (RTA) due to toluene toxicity was made. Urinary L-FABP levels decreased as the condition improved.

Urinary L-FABP is a practical and rapid diagnostic and follow-up tool for toluene-induced RTA, and it is helpful to measure it in addition to conventional methods.

## Introduction

Painters need to be careful about toluene and xylene contained in solvent-based paints. Poisoning symptoms occur if proper working conditions and respiratory protection are not used. Toluene poisoning includes acute poisoning and chronic poisoning. Toluene poisoning is characterized by rhabdomyolysis, flaccid quadriplegia, and renal tubular acidosis (RTA) [1]. Acute poisoning causes psychotic symptoms such as restlessness and euphoria [2]. RTA of toluene toxicity causes proximal and distal tubular damage [1,3,4]. Traditionally, RTA has been diagnosed by ammonium chloride and bicarbonate stress tests. However, the testing method is complex, and it is not a test that can be easily performed in many facilities [5]. In this case, urinary liver-type fatty acid-binding protein (L-FABP) was used to diagnose and track RTA associated with chronic toluene poisoning. Urinary L-FABP is a biomarker that reflects renal tubular damage [6]. Therefore, measuring urinary L-FABP in addition to conventional diagnostic and follow-up methods is helpful, and we summarize its clinical characteristics.

## Case presentation

The patient is a 54-year-old man. On admission, the patient was unconscious, and his medical history was unknown. After admission, the patient regained consciousness and had a history of hypertension and high uric acid levels. He was involved in a traffic accident the day before and was taken to another hospital, but no abnormalities were detected, and he returned home. While driving for the second day in a row, he crashed into a power pole without being prompted. He was transported by ambulance with unconsciousness and bruises all over his body. As a result of the examination, it was found that the patient had consciousness disturbance due to GCS E3V4M5. His temperature was 37.1°C, blood pressure was 152/100 mmHg, pulse rate was 93 beats/min, respiratory rate was 18 breaths/min, and SpO_2_ was 97% (on room air). He had pain and bruising on his right upper extremity and tenderness and swelling in the midline of his anterior chest. There was no damage to his pelvis or lower legs, except for abrasions to his abdomen. There was paint on both his clothes, forearms, and fingers. He underwent chest X-ray and head and thoracoabdominal CT, but no abnormal findings due to trauma were found. Blood tests revealed hyperuricemia, decreased renal function, and hyperkalemia. Blood gas analysis showed metabolic acidosis with a normal anion gap (AG). Qualitative urine findings were pH of 5.5, proteinuria of (2+), and occult blood of (2+) (Table 1).

**Table 1:** Laboratory results upon admission CRP, C-reactive protein; TP, total protein; Alb, albumin; AST, aspartate aminotransferase; ALT, alanine aminotransferase; LDH, lactate dehydrogenase; γ-GTP, γ-glutamyl transpeptidase; T-Bil, total bilirubin; BUN, blood urea nitrogen; eGFR, estimated glomerular filtration; WBC, white blood cell; RBC, red blood cell; Hb, hemoglobin; Ht, hematocrit; Plt, platelet; BE, base excess; SG, specific gravity; NAG, N-acetyl-β-D-glucosaminidase; β2MG, β2-microglobulin; L-FABP, L-type fatty acid-binding protein

Test	Result	Unit	Reference range
CRP	0.03	mg/dL	<0.3
TP	9.3	g/dL	6.7-8.3
Alb	5.3	g/dL	3.8-5.2
AST	28	IU/L	10-40
ALT	25	IU/L	5-45
LDH	265	U/L	124-222
γ-GTP	33	IU/L	<30
T-Bil	0.5	mg/dL	0.2-1.2
BUN	20.9	mg/dL	8.0-20.0
Creatinine	1.95	mg/dL	0.47-0.79
eGFR	30	mL/min/1.73m^2^	>60
Sodium	141	mEq/L	137-147
Potassium	4.3	mEq/L	3.5-5.0
Chlorine	118	mEq/L	98-108
Glucose	116	mg/dL	70-109
HbA1c	5.8	％	4.7-6.2
WBC	6900	/μL	3300-9000
RBC	468	×10^4^/μL	430-570
Hb	14.3	g/dL	13.5-17.5
Ht	44.9	44.9%	39.7-52.4
Plt	31.0	10^4^/μL	14-34
Blood gas analysis			
pH	7.208		7.35-7.45
pCO_2_	33.9	mmHg	35-45
pO_2_	80.5	mmHg	80-100
HCO_3_^-^	13.2	mEq/L	23-28
BE	‐13.6	mEq/L	-2.2*±*1.2
Lactate	1.23	Mmol/L	<1.3
Anion gap	9.8	mEq/L	10-14
Urinalysis			
pH	5.5		5.0-8.0
SG	1.028		1.010-1.025
Glucose	(-)		(-)
Protein	(2+)		(-)
Occult hematuria	(2+)		(-)
Hippuric acid	7.52	g/L	<1.0g/L
Methylhippuric acid	≤0.01	g/L	<0.5g/L
Urinary NAG	3.4	U/L	<11.5
Urinary β2MG	14860	μg/L	<200
Urinary L-FABP (creatinine correction)	22.1	μg/g･Cr	<8.4

**Figure 1:**
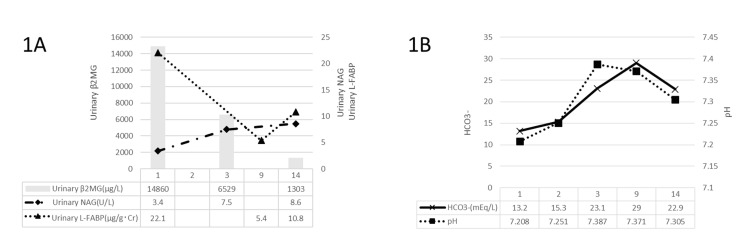
(1A and 1B) Progress after hospitalization After admission, acidosis, urinary L-FABP, and urinary β2MG improved. On the ninth day of onset, it was confirmed that the blood pH was within the normal range, there were no symptoms, and the patient was allowed to return to work. Even after returning to work, the blood HCO_^3^_^-^ concentration remained within the normal range, with no symptoms. pH indicated arterial blood but on day 14 it was venous. Venous blood has a higher CO_2_ value than arterial blood. This affects pH. β2MG, β2-microglobulin; L-FABP, L-type fatty acid-binding protein; NAG, N-acetyl-β-D-glucosaminidase

The electrocardiogram showed sinus rhythm. Toluene poisoning was suspected based on the presence of impaired consciousness, occupation is a painting job that involves toluene, and the presence of metabolic acidosis of the normal AG. His urine hippuric acid levels were measured and was admitted to the hospital. He was started on fluid replacement with Ringer's acetate solution, but the acidosis was only mildly corrected after 2 L administration. Normal AG metabolic acidosis was considered to be RTA. Urinary L-FABP, urinary N-acetyl-β-D-glucosaminidase (NAG), and urinary β2-microglobulin (β2MG) were measured to evaluate the degree of injury and site. The progress is shown in Figure 1.

Urinary NAG was within the normal range, but urinary β2MG and urinary L-FABP were elevated, so a diagnosis of proximal tubular disorder was made. At the same time, a bicarbonate stress test was performed to diagnose renal tubular disorders. However, the rapid HCO_3_^-^ correction caused phlebitis, reducing the dripping rate from 100mL/h to 150mL/h. As a result, the bicarbonate stress test could not achieve the target blood HCO_^3^_^-^ concentration of 28 mmol/dL. Furthermore, it was discovered that urinary HCO_3_^-^ could not be measured at our facility, so we abandoned the challenge test itself. On the second day of hospitalization, the patient started complaining of joint and muscle pain in his limbs, and his consciousness improved. As his consciousness improved, the patient revealed that he was in the painting business, driving a commercial vehicle with paint in it, and that the vehicle's interior was constantly filled with the odor of paint. An increase in urinary hippuric acid was later confirmed, and the patient was diagnosed with proximal tubular acidosis and rhabdomyolysis due to chronic toluene toxicity. Since there was no exposure to toluene due to hospitalization, symptoms improved with only symptomatic treatment. Improvements in acidosis, urinary L-FABP, and urinary β2MG were also observed. Head MRI revealed no leukoencephalopathy, and the patient was discharged without sequelae on the fifth day of hospitalization. After being discharged from the hospital, he did not resume work to avoid exposure to toluene. At an outpatient examination, the patient was allowed to return to work on the ninth day after the onset of symptoms, as his blood pH was within the normal range and he had no signs. When returning to work, they were instructed to improve car ventilation, take appropriate breaks during work hours, and remove as much paint as possible from their bodies. After returning to work on the 14th day of onset, the HCO_3_^-^ correction remained within the normal range, with no symptoms. Urinary β2MG was expected, but urinary L-FABP was slightly elevated, so we instructed him to follow the precautions and have an organic solvent health check every year.

## Discussion

Organic solvents in the painting industry can cause poisoning without proper working conditions and respiratory protection. For this reason, it is necessary to be careful about toluene and xylene, which contain solvent-based paints. Toluene poisoning is often observed in Japan [7]. If ingested orally, gastrointestinal symptoms such as vomiting and diarrhea will occur. Acute inhalation causes respiratory symptoms such as chemical pneumonitis and acute respiratory distress syndrome. In addition, central nervous system (CNS) symptoms such as coma and seizures and cardiovascular symptoms such as tachyarrhythmia are observed. On the other hand, the toxic symptoms caused by chronic toluene exposure are mainly CNS symptoms [8]. It exhibits a variety of symptoms, including abnormal behavior, memory impairment, and ataxia. Irreversible leukoencephalopathy with cerebral and cerebellar atrophy may occur. Non-CNS disorders are often accompanied by systemic muscle weakness due to RTA, hypokalemia, and rhabdomyolysis [9,10]. RTA involves damage to the proximal or distal renal tubules [3]. This case was diagnosed as chronic toluene poisoning due to the toluene exposure environment, symptoms, increased urinary hippuric acid concentration, and RTA. In cases of chronic toluene poisoning that causes CNS symptoms, it is difficult to obtain a patient's medical history, and the presence of normal AG metabolic acidosis without diarrhea is considered to be a suspicious finding.

Traditionally, renal tubular damage due to chronic toluene poisoning was diagnosed by evaluating the reabsorption capacity of the proximal tubule using a bicarbonate stress test and the ability of the distal tubule to convert to uric acid using an ammonium chloride stress test [11]. There were only seven reports of toluene poisoning due to industrial accidents in Japan in the five years from 2015 to 2019 [7]. Therefore, there are few opportunities for these tests to be conducted due to the small number of cases. As a result, bicarbonate and ammonium chloride tests for chronic toluene poisoning are not blood and urine tests that clinicians can perform at any facility. Among these, bicarbonate stress tests require frequent blood gas tests and special urine collections, and the number of facilities that can measure the HCO_^3^_^-^ concentration in urine is limited [5]. Urinary L-FABP is released into the urine from the proximal renal tubule and increases in response to ischemia and oxidative stress injury. It is a marker that reflects the results of renal tubular damage and is said to detect renal tubular impairment sensitively. It is a simple, non-invasive test that uses a few drops of urine and provides results within a few hours. For this reason, it has been widely used in recent years to predict the progression and prognosis of renal damage in acute kidney injury and chronic kidney disease [12]. It was used for diagnosis and follow-up observation in this case as well. Urinary NAG did not increase at admission due to renal tubular damage, but urinary β2MG and L-FABP increased. Over time, symptoms improved with improvement in pH, urinary β2MG, and urinary L-FABP. In addition, after being discharged from the hospital and resuming painting work, urinary NAG and urinary β2MG were expected. Still, a slight increase in L-FABP was observed, which may reflect re-exposure to toluene. Typically, urinary β2MG is filtered in the glomerulus and then mostly reabsorbed in the proximal renal tubule. Therefore, it is not excreted in the urine. However, if the renal tubules are damaged, they cannot be reabsorbed and are excreted in the urine. Urinary NAG is particularly abundant in proximal tubular epithelial cells. Typically, it is not passed in the urine, but when the proximal tubule is injured, urinary NAG deviates from the proximal tubular epithelial cells and is excreted in the urine. In summary, urinary β2MG is used as an indicator of functional disorder of the proximal tubule, and urinary NAG is used as an indicator of organic disorder of the proximal tubule. It is suggested that renal tubular damage caused by chronic toluene poisoning may cause functional but not organic damage. Camara-Lemarroy et al. have also reported that renal tubular damage caused by toluene poisoning is due to renal tubular dysfunction due to HCO_3_^-^ [1]. However, urinary β2MG and urinary NAG are unstable due to changes in urinary pH, so their accuracy is considered insufficient [13]. The urinary pH of chronic toluene-poisoned urine is dangerous due to RTA, and the urinary L-FABP reflects the result of renal tubular damage. This is for the sensitive response to the case's progress.

Urinary β2MG and urinary L-FABP are tests that can provide measurement results within a few hours. Urinary β2MG and urinary L-FABP may be more beneficial than bicarbonate stress tests or ammonia stress tests to diagnose and follow-up with chronic toluene poisoning. Urinary L-FABP shows a more sensitive response than urinary β2MG. As in this case, these renal tubular markers decreased when the patient avoided toluene exposure and increased when he resumed work in the painting industry. Tests commonly used in the clinical course of toluene poisoning include evaluating potassium levels using electrocardiograms, blood gas analysis, qualitative urine tests, and blood biochemical tests [8]. However, if urine alone can determine the extent of renal tubular damage and can be used to monitor the clinical course of chronic toluene poisoning, it could be a substitute for existing tests.

Because the number of cases is small and the disease is toxic, it is challenging to conduct clinical trials for chronic toluene poisoning. However, since it is a non-invasive test that may be used for outpatient follow-up and periodic examinations for occupational exposure, it is necessary to accumulate more cases.

## Conclusions

Painters must be careful of poisoning symptoms caused by toluene and xylene in solvent-based paints. Chronic toluene poisoning primarily causes central nervous system symptoms, RTA, and rhabdomyolysis. In this patient's case, urinary β2MG and L-FABP measurements were suggested to be useful for early diagnosis and follow-up. These non-invasive tests may be helpful for follow-up of toluene poisoning and occupational exposure testing.
